# Report of Cases in Dr. Hamilton’s Surgical Dispensary, in the Medical Department of the University of Buffalo

**Published:** 1848-07

**Authors:** Wm. White


ART. II.—*Report of Cases in Dr. Hamilton's Surgical Dispensary, in the Medical Department of the University of Buffalo.*

The case of Hare-lip, (case 7) reported in the May number of the last volume of this Journal, has had a singular termination. Dr. H. supposed that the parents understood that they were to return with the child and have the lip dressed in a day or two; but they did not so understand it, and for several weeks, notwithstanding the most diligent enquiry, nothing could be heard of them; and the conclusion was that the child had died from the operation. It is now however ascertained that the little patient is alive and well, and that the lip is handsomely united. Yet the dressings were never touched or removed and the stitches were allowed to fall out spontaneously.

*Necrosis of Inferior Maxilla.*—James McKay aged 4 years. Had Rubeola eleven months since, about three months after which a pain commenced in the lower jaw, or in a tooth, followed by a swelling.

The jaw is now considerably distorted, and the teeth upon that side are loosening. Patient is in pretty good health, and has very little pain.

The parents were advised not to allow any interference in the process of nature to exfoliate the dead bone at present. If the general health should suffer, or the exfoliation proceed too slowly, both medical and surgical interference would be proper. The class were cautioned especially against the *nimia diligentia*, of officious surgery in similar cases. It is seldom that here anything more is necessary, than to wait patiently the course of nature. It is well enough to inject a weak solution of castile soap and water, merely to keep the sore cleanly, but stimulating and irritating injections are positively injurious.

May 15th, *Encysted Tumor.*—Man aged 70 years. Sixty years ago this tumor was first seen on his left temple. It has grown gradually to about three inches in length, and one and a half in breadth. It was at first elastic and has never been painful. Within a few weeks it has become much softer, and probably will open. Of course even a simple tumor of such long existence, and showing a change of character at this age, is not without danger. But he is advised to let it open, and perhaps it will discharge and disappear, if it does not, it will then be the proper time to interfere. It has illustrated one thing, namely: how little disposed such tumors are to disappear spontaneously, and it may hereafter illustrate another, namely: that they may at last become malignant.

The same patient has a chronic inflamation of the capsule of the knee joint of five years standing. The limb is improved by moderate use. This is not unusual.

*Double Inguinal Hernia.*—Lad seven years old; never has been able to keep the hernia up. Dr. H. applied Chase's truss, which after several weeks trial has succeeded perfectly.

*Deafness, &c.*—Negro aged about 20. This, if stated correctly by the negro, is a singular case. He appears very intelligent and honest.

Five years ago he lost his left eye from inflammation following an arrow wound, and as the inflammation in the eye subsided, his hearing became defective; and finally when the inflammation was fully gone, he was completely deaf. The inflammation in his eye, however, returned, and with it, to a considerable extent, his hearing. These alternations have taken place several times, and the two circumstances always bear the same relation to each other. In some of these attacks of deafness, he has had much pain in the ear and discharge of matter, he had also severe pain in his head. For the last year he has been perfectly deaf without any intervals of partial relief.

It is diagnosed as a destruction of the labarynth by inflammation and suppuration, and is consequently irremediable.

*Chronic Synovitis.*—A man from Oswego Co. aged about 30, sprained his knee two years since. It immediately became much swollen and it has remained lame and more or less swollen ever since. He has derived temporary benefit from iodine applied externally, and also from a succession of blisters applied by Dr. Hard of Oswego. He has tried perfect quiet for ten days, but at the end of this time the limb was more tender than at first.

Dr. H. after alluding to the diagnostic marks in this case, and advising a continuance of the iodine externally, with the addition of the cold dash and moderate use of the limb, remarked upon the frequency with which he had noticed that moderate use of the limb had restored the joint to health. It is a somewhat popular professional error to keep such limbs a long time perfectly still. This is certainly proper when recently injured, or acutely inflamed; and inasmuch as frequently the swelling and tenderness continue scarcely abated while the inflammation has lost entirely its active character, it is certainly not singular that the change should not be always noticed and the treatment accordingly varied. There are two or three points however, which may serve as our guides. If several weeks or months have elapsed since the receipt of the injury; if the swelling is rather œdematous than otherwise, or extends below considerably beyond the diseased part, if the tenderness is also spreading over the whole limb, and is superficial, if the patient is of lax habit, or if he assures you that it feels better when in moderate use; if several or all of these conditions exist you may feel certain that it requires the tonic of action, and the longer it is kept still the worse will matters become. (The patient has been heard from several weeks since the above was written, and he is rapidly improving.)

*Partial Luxation of Acromial end of Clavicle Upward.*—This is an old lady who fell on the shoulder last christmas, and produced a semi-luxation of the outer end of the clavicle. She has also suffered considerably ever since from lameness in the deltoid, soreness in the joint and pain and numbness in the hand and arm. It is for these latter difficulties, which threaten to render her arm useless, that she applies for relief. The luxation has nothing to do with these circumstances, and is only of interest as being of rare occurrence. The luxation is in that direction in which it almost always occurs, viz, upwards. It is too late now to think of reduction. The means of reduction and retention are precisely those which would be proper in a *fractured* clavicle. But it is not probable that if the proper apparatus had been applied from the first it could have been made to stay in place. Nothing has yet been said in the books of the difficulty of retaining the small articular surfaces of long bones in their places, yet it is very important for you to know that it is nearly impossible. This remark applies to dislocations of the clavicle at either end, to dislocation of the lower end of the ulna, of the upper and lower end of the fibula, and to some extent to dislocations of the upper end of the radius; it also applies in some degree to phalangeal and metacarpo-phalangeal dislocations. Of all these the clavicle and lower end of the ulna are the most troublesome. If you will measure the breadth and circumference of this latter after a heavy fall on the palm of the hand, producing what is called a sprain of the wrist, you will often find that the ulna is separated from the radius and generally carried a little forward, and so it will always remain.

*Sub-luxation of last Articulation of Thumb.*—This is another illustration of what has just been remarked. The young man fell four weeks since upon the distal end of his thumb. It was followed by some inflammation and now a slight deformity, evidently produced by a partial displacement of the articular surface, is apparent. There also remains slight anchylosis.

*Compound Comminuted Fracture of Humerus.*—This gentleman is a private patient of Drs. Sprague and Hamilton, but he has consented to give the class the benefit of his misfortune. Fourteen weeks since his arm was broken near the condyles by a severe blow from some part of the waggon with which a couple of horses were running. It was dressed by Drs. Hamilton & Sprague, by removing the loose fragments—applying moderately, as a support, an angular splint with moveable joint, and then laying it on pillows. The inflammation has gradually subsided, but the wound still continues to discharge, and there is now very little motion in the joint. This however is perceptibly increasing, and Dr. H. hopes he yet may recover the use of his limb entirely. This however he has never been encouraged to expect.

*Congenital Encysted Tumor.*—This child, aged fourteen months, has a large translucent tumor on right side of neck—the size of my fist, which tumor commenced prior to birth. As its appearance was extraordinary, Dr, H. opened it carefully with an iris knife and expressed the contents, which were a pale serum, resembling the fluid of a hydrocele. Dr. H. directed that this operation should be repeated as often as it filled, and he predicted that after a time it would imperfectly suppurate, and disappear.

*Strabismus.*—Miss ——, aged 18, of Buffalo. The right eye is turned in very much. Produced by small pox, when she was two years old. Five years since she was operated upon by an itinerant at Milwaukee Wis., but the operation left the eye just as it was before.

Dr. H. operated before the class upon the same eye, and found the tendon inserted about two lines farther back than usual; this accounts for the operator not reaching the tendon before. The eye immediately became straight after the tendon was divided, and still remains so.

WM. WHITE.

